# Effects of Endobacterium (*Stenotrophomonas maltophilia*) on Pathogenesis-Related Gene Expression of Pine Wood Nematode (*Bursaphelenchus xylophilus*) and Pine Wilt Disease

**DOI:** 10.3390/ijms17060778

**Published:** 2016-05-25

**Authors:** Long-Xi He, Xiao-Qin Wu, Qi Xue, Xiu-Wen Qiu

**Affiliations:** 1 Co-Innovation Center for Sustainable Forestry in Southern China, College of Forestry, Nanjing Forestry University, Nanjing 210037, China; longxihe@163.com (L.-X.H.); xq2159@163.com (Q.X.); 2 Jiangsu Key Laboratory for Prevention and Management of Invasive Species, Nanjing Forestry University, Nanjing 210037, China; 3 Jiangxi Forest Pest Control and Quarantine Bureau, Nanchang 330038, China; 4 Poyang Lake Eco-economy Research Center, Jiujiang University, Jiujiang 332005, China; qiuxiuwen3@163.com

**Keywords:** transcriptome, differentially expressed genes, bacteria, pine, *Bursaphelenchus xylophilus*

## Abstract

Pine wilt disease (PWD) caused by the pine wood nematode (PWN), *Bursaphelenchus xylophilus*, is responsible for devastating epidemics in pine trees in Asia and Europe. Recent studies showed that bacteria carried by the PWN might be involved in PWD. However, the molecular mechanism of the interaction between bacteria and the PWN remained unclear. Now that the whole genome of *B. xylophilus* (*Bursaphelenchus xylophilus*) is published, transcriptome analysis is a unique method to study the role played by bacteria in PWN. In this study, the transcriptome of aseptic *B. xylophilus*, *B. xylophilus* treated with endobacterium (*Stenotrophomonas maltophilia* NSPmBx03) and fungus *B. xylophilus* were sequenced. We found that 61 genes were up-regulated and 830 were down-regulated in *B. xylophilus* after treatment with the endobacterium; 178 genes were up-regulated and 1122 were down-regulated in fungus *B. xylophilus* compared with aseptic *B. xylophilus*. Gene Ontology and Kyoto Encyclopedia of Genes and Genomes analyses were used to study the significantly changed biological functions and pathways for these differentially expressed genes. Many pathogenesis-related genes, including glutathinone *S*-transferase, pectate lyase, ATP-binding cassette transporter and cytochrome P450, were up-regulated after *B. xylophilus* were treated with the endobacterium. In addition, we found that bacteria enhanced the virulence of PWN. These findings indicate that endobacteria might play an important role in the development and virulence of PWN and will improve our understanding of the regulatory mechanisms involved in the interaction between bacteria and the PWN.

## 1. Introduction

Pine wilt disease (PWD) is a worldwide forest disease affecting several species of pine trees (*Pinus* spp.), and it has been epidemic in Asia (Japan, China, and Korea) and in Portugal [1,2,3,4]. In these countries, PWD has killed a large number of pine trees and caused serious economic losses and ecological damages [5]. The pine wood nematode (PWN), *Bursaphelenchus xylophilus* [6], is considered the causal pathogenic agent of PWD and is designated an important quarantine species [7]. The PWN invades healthy pines via sawyer beetles (*Monochamus* spp.), particularly *Monochamus alternatus* during feeding or oviposition [8,9]. After PWNs invade the trees, they move inside the host rapidly via cambium, axial and radical parenchyma, and feeding upon living tissues, which ultimately results in complete wilting of pine trees.

Recent studies reported that the PWN carries diverse bacterial communities and bacteria may play a crucial role in PWD development [10,11,12,13]. Some bacteria isolated from *B. xylophilus* could produce toxins, which were suggested to cause pine tree wilting [10,14]. Several studies revealed that bacteria associated with *B. xylophilus* can also secrete a cellulose degradation enzyme, which is extremely important for PWN colonization [15,16]. The main bacterial genera associated with PWN are *Bacillus*, *Brevibacteria*, *Burkholderia*, *Pseudomonas*, *Serratia*, and *Stenotrophomonas* [17,18,19,20]. The PWN-associated bacteria are primarily on the body surface of nematodes. Additionally, PWNs from different countries and regions carry distinct genera of bacteria; the nematode could acquire and carry random bacteria from soil, pines, and vectors [17,18,19,20]. Many details of the relationship between bacteria and PWNs are still uncertain due to the complexity of the PWN surface coat.

Wu *et al.* [13] isolated 15 species of endobacteria from ten different strains of the PWN from Anhui, Jiangsu, Zhejiang, Guangdong, Yunnan, and Hubei Provinces, China. The dominant genera of endobacteria isolated from PWNs were *Stenotrophomonas*, *Achromobacter*, *Ewingell*a, *Leifsoni*a, *Rhizobium*, and *Pseudomonas* [13]. A recent study showed that endobacteria might play a role in the development and virulence of the PWN, *i.e.*, *Achromobacter xylosoxidans* ss. *xylosoxidans* NSBx.22 from PWN AA3 with low virulence could slow the nematode growth on *Botrytis cinerea* and enhanced the virulence of PWN AA3 after pine seedlings were infected [21]. It was also found that the community structure of endobacteria from *B. xylophilus* was very similar among high virulence *B. xylophilus*, low virulence *B. xylophilus*, and *B. mucronatus* [22]. Xiang *et al.* [23] found that *Stenotrophomonas*, Pseudomonadaceae (unclassified) or Rhizobiaceae (unclassified) were predominant in the bacteria communities associated with virulent *B. xylophilus* and non-virulent *B. mucronatus*. It would be beneficial to study the mechanism of interaction between endobacteria and the PWN and the effect of bacteria on gene expression of *B. xylophilus*.

The plant cell wall comprises mainly pectin, cellulose, and hemicellulose, which are the primary barriers to pathogens and parasites [24]. Researchers cloned cellulose and pectin lyase genes from the PWN, and speculated that these hydrolase genes were closely related to pathogenicity of the PWN [25]. Pine trees generate numerous secondary metabolites to combat invasion by the PWN. Therefore, degrading these secondary metabolites has become an important factor in successful infection of the host by the PWN. The mechanism of the bacteria assisting PWNs to parasitize hosts has become a research focus for understanding the role of bacteria in PWD. RNA sequencing (RNA-seq) technology provides an effective method to investigate the expression level of messenger RNA (mRNA) under different ecological conditions and at different developmental stages. Daurelioet *et al.* [26] found that 316 transcript-derived fragments showed differential expression in tobacco after infection with *Xanthomonas axonopodis* pv. *citri* by transcriptome analysis. Kikuchi *et al.* [27] first published the whole genome of *B. xylophilus*. These previous results provided a good basis for analyzing the transcriptome of the PWN.

The objective of the present study is to elucidate the effects of bacteria on expression of genes related to the pathogenicity of the PWN using transcriptome analysis. These results will help us to further understand the nature of the relationship between bacteria and the PWN at the molecular level.

## 2. Results

### 2.1. Mapping of RNA-Seq Reads in the Genome of B. xylophilus

mRNA was sequenced from aseptic PWNs, PWNs treated with endobacterium (*S. maltophilia* NSPmBx03), and fungus PWN using paired end protocols on an Illumina HiSeq^TM^2000 system (Shenzhen, China). A total of 143,978,136 reads, 12.96 Gbp, and 17,286 genes were obtained from clean reads of three different treatments (Table S1). Approximately, 75.2% and 72.1% of our reads were mapped to the reference genome and PWN genes, respectively. Over 70% of our reads were uniquely mapped reads.

### 2.2. Differentially Expressed Genes in Transcriptome Sequence of PWNs Treated with Endobacterium and Fungus PWNs

Differentially expressed genes (DEGs) between aseptic PWNs and PWNs treated with *S. maltophilia* NSPmBx03 were examined. Compared to aseptic PWNs, a total of 891 significant DEGs were identified with at least a two-fold change at the expression level and false discovery rate (FDR) <0.001. Of these genes, 61 were up-regulated and 830 down-regulated. A total of 1300 genes were identified as DEGs between aseptic PWNs and fungus PWNs. Of these genes, 178 were up-regulated and 1122 down-regulated. The quantity of DEGs in PWNs treated with one endobacterial strain was less than that in fungus PWNs. The number of DEGs between PWNs treated with endobacterium and fungus PWNs was 625, which was less than that between aseptic PWNs and fungus PWNs (Figure S1). These results implied that bacteria affected expression of some genes of the PWN.

### 2.3. Functional Annotation and Classification for DEGs of the PWN

**Figure 1:**
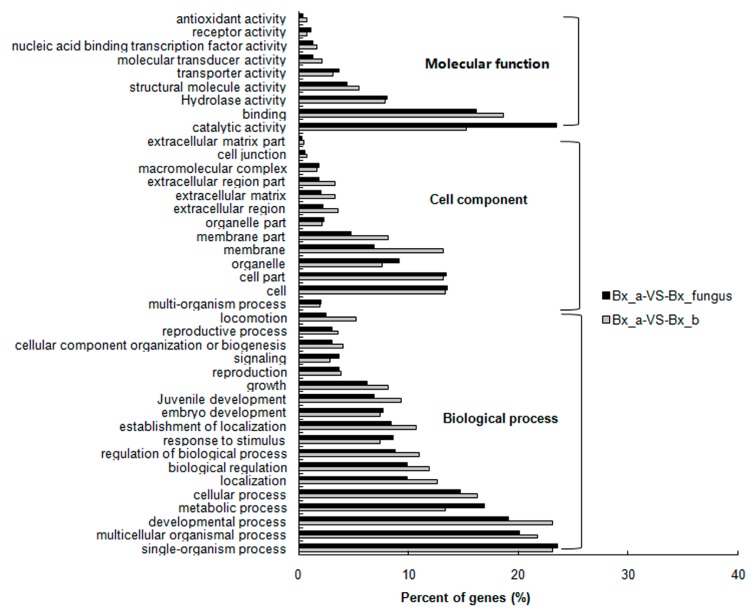
Gene Ontology (GO) terms for the transcriptome sequences derived from differently treated *B. xylophilus* (*p* < 0.05). Gray bars indicate significantly enriched GO terms in endobacterium-treated PWN samples (Bx_b) compared to aseptic PWNs (Bx_a); black bars indicated significantly enriched GO terms in fungus PWNs (Bx_fungus) compared to aseptic PWNs (Bx_a).

Gene Ontology (GO) analysis was used to search for significantly enriched GO terms of DEGs. The main GO categories included molecular function, cellular component, and biological process. Compared to aseptic PWNs, the “catalytic activity” (GO:0003824), “binding” (GO:0005488), “hydrolase activity” (GO:0016817), “structural molecule activity“ (GO:0005198), and “transporter activity” (GO:0005215) categories were among the top molecular function terms in both PWNs treated with NSPmBx03 and fungus PWNs (Figure 1). In the cell component category, most DEGs were involved in the “cell” (GO:0005623), “cell part” (GO:0044464), “organelle” (GO:0043266), “membrane” (GO:0016020), and “membrane part” (GO:0044425). In addition, “single-organism process” (GO:0044699), “developmental process” (GO:0003006), “multicellular organismal process” (GO:0032501), and “metabolic process” (GO:0008152) were the top categories enriched in the biological process. In general, the aforementioned GO terms accounted for the majority of the DEGs. Furthermore, some GO terms play an important role in PWN development, such as embryo development, juvenile development, regulation of growth rate and hydrolase activity, in response to external stimuli.

### 2.4. Analysis of Cell Wall Degradation-Related Genes

**Figure 2:**
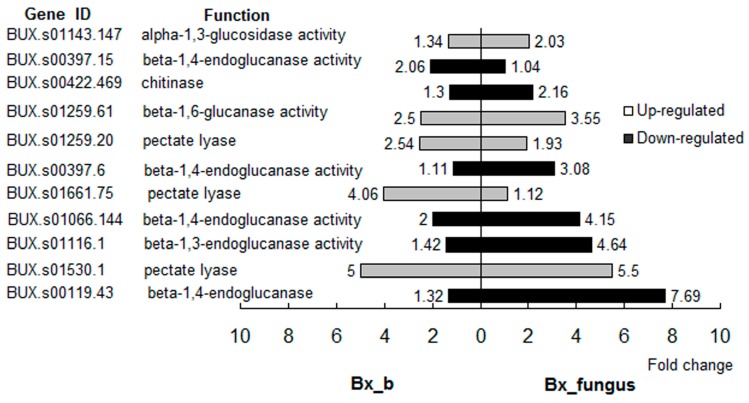
Putative cell wall hydrolase genes in *B. xylophilus* treated with endobacterium and fungus *B. xylophilus* (more than two-fold change in *B. xylophilus* treated with endobacterium or wild-type *B. xylophilus*). Bx_b: *B. xylophilus* treated with endobacterium, Bx_fungus: fungus *B. xylophilus*.

Enzymes of cell wall degradation are intrinsically related to nematode pathogenicity in host pines [25]. Compared with aseptic PWNs, the expression levels of six cell wall degradation-related genes changed significantly (over two-fold) in PWNs treated with *S. maltophilia* NSPmBx03. Of these DEGs, three pectate lyase genes (*BUX.s01259.20*, *BUX.s01661.75*, and *BUX.s01530.1*) and one cellulase gene *(BUX.s01259.61*) were up-regulated over two-fold (Figure 2). In fungus PWNs, eight cell wall degradation-related genes were DEGs (over two-fold). The expression of *BUX.s01530.1* was up-regulated 5.5-fold. However, expression levels of cellulase genes were significantly down-regulated (Figure 2). These results indicated that endobacteria could affect the expression levels of cell wall degradation-related genes of PWNs and these genes changed much more evidently in fungus PWNs.

### 2.5. Detoxification-Related Gene Analysis

**Figure 3:**
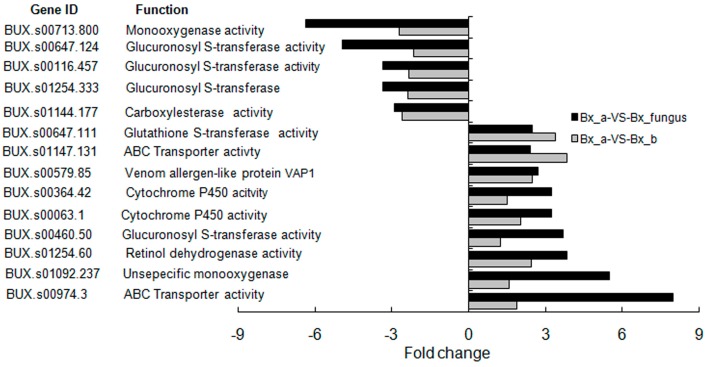
Putative detoxification-related genes most differentially expressed, with higher levels in *B. xylophilus* treated with *S. maltophilia* NSPmBx03 and fungus *B. xylophilus*. Bx_a: aseptic *B. xylophilus*, Bx_b: *B. xylophilus* treated with *S. maltophilia* NSPmBx03, Bx_fungus: fungus *B. xylophilus*. The left of the vertical axis indicates down-regulated genes, and the right indicates up-regulated genes.

The capacity for detoxification is a key factor in PWN colonization of pines [27]. Compared with aseptic PWNs, the expression level of several detoxification-related genes changed significantly in PWNs treated with *S. maltophilia* NSPmBx03 and fungus PWNs, and their functions included glutathinone *S*-transferase (*GST*) activity, ATP-binding cassette (*ABC*) transporter, venom allergen-like protein VAP1, rethinol dehydrogenase, cytochrome P450 (*CYP*), and carboxylesterase (Figure 3). *GST* and *CYP* genes were primary DEGs related to detoxification. These results suggested that the endobacterium and other bacteria genera have an important role in regulation of detoxification-related gene expression, especially *CYP* and *GST*. The expression level of *BUX.s01147.131* (*ABC* transporter) and *BUX.s00647.111* (*GST*) were up-regulated over three-fold in PWNs treated with NSPmBx03. The expression of *BUX.s00974.3* (*ABC* transporter) and *BUX.s01092.237* (*CYP*) were up-regulated 8-fold and 5.5-fold in wild-type PWNs, respectively. In addition, the expression levels of *BUX.s01147.131* and *BUX.s00647.111* were more greatly up-regulated in PWN treated with NSPmBx03 than in fungus PWNs. These results indicated that the bacteria had an important function in the expression of these detoxification-related genes of PWN.

### 2.6. Reproduction-Related Gene Analysis

**Figure 4:**
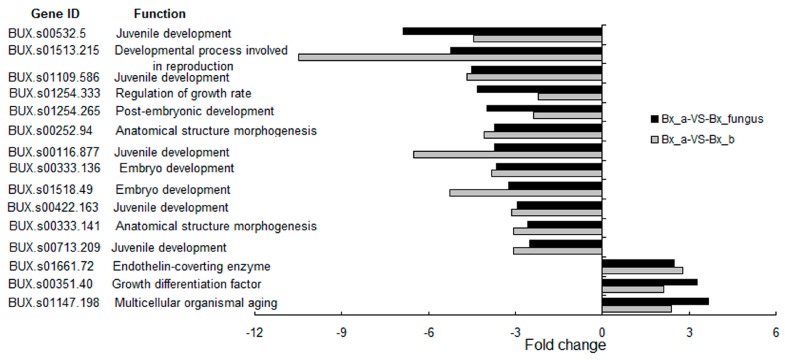
Putative reproduction-related genes in *B. xylophilus* treated with endobacterium and fungus *B. xylophilus* (fold-change > 2). Bx_a: aseptic *B. xylophilus*, Bx_b: *B. xylophilus* treated with *S. maltophilia* NSPmBx03, Bx_fungus: fungus *B. xylophilus.* The left of the vertical axis indicates down-regulated genes, and the right indicates up-regulated genes.

A number of reproduction-related genes were differentially expressed in PWNs treated with *S. maltophilia* NSPmBx03 and fungus PWNs compared with aseptic PWNs (Figure 4). The functions of reproduction-related genes were involved with juvenile development, embryo development, anatomical structure morphogenesis, endothelin-converting enzyme, growth differentiation factor, developmental process involved in reproduction, regulation of growth rate, and multicellular organismal aging. Most juvenile and embryo development-related genes were down-regulated. The expression level of *BUX.s01513.215* (developmental process involved in reproduction) and *BUX.s00116.877* (juvenile development) were down-regulated over six-fold in PWNs treated with NSPmBx03, and the expression of *BUX.s00532.5* (juvenile development) was down-regulated over six-fold in fungus PWNs. These results suggested that PWN-associated bacteria could slow the growth of PWN.

### 2.7. Kyoto Encyclopedia of Genes and Genomes (KEGG) Pathway Enrichment Analysis

**Figure 5:**
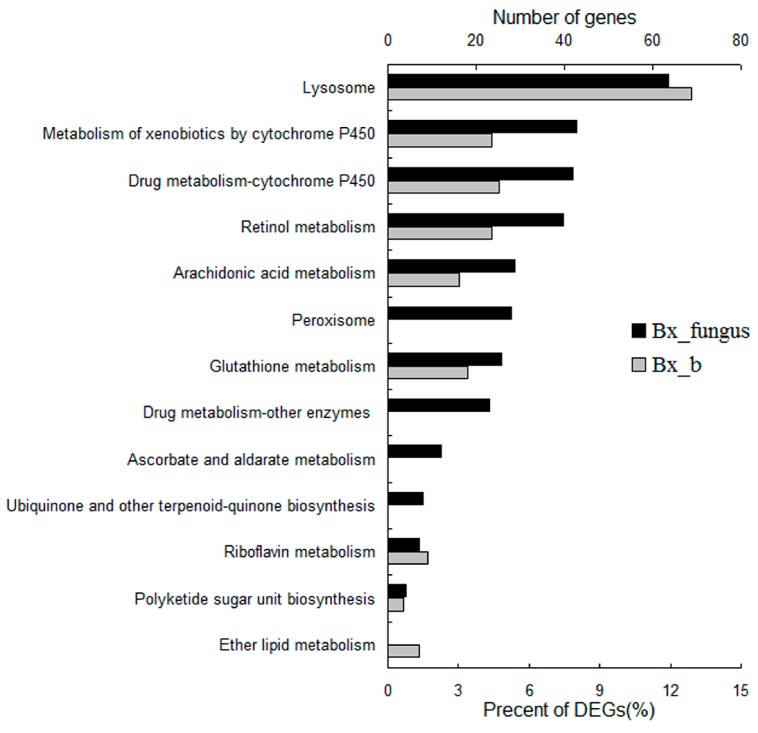
Relative pathways significantly enriched in differentially expressed genes (DEGs) of PWNs treated with endobacterium and fungus PWNs (*p* < 0.005). DEGs were enriched in detoxification pathways and turpentine-related metabolism pathway. Bx_b: *B. xylophilus* treated with *S. maltophilia* NSPmBx03, Bx_fungus: fungus *B. xylophilus.*

The pathway enrichment for DEGs was performed using KEGG. From the transcriptome of PWNs treated with *S. maltophilia* NSPmBx03, 75 DEGs were involved in detoxification pathways containing drug metabolism cytochrome P450, metabolism of xenobiotics by cytochrome P450, lysosome, and glutathione metabolism (*p* < 0.05) (Figure 5). Twenty-eight genes were enriched in turpentine-related metabolism including ether lipid metabolism, arachidonic acid metabolism, polyketide sugar unit biosynthesis, and retinol metabolism. These metabolic pathways may be important for the PWN to parasitize pines. In fungus PWNs, 238 genes (28.4%) were enriched in detoxification pathways containing lysosome, metabolism of xenobiotics by cytochrome P450, drug metabolism cytochrome P450, peroxisome, glutathione metabolism, drug metabolism-other enzymes, ascorbate and aldarate metabolism. Fifty-nine genes were involved in turpentine-related metabolism containing arachidonic acid metabolism, rethinol metabolism, ubiquinone and other terpenoid-quinone biosynthesis, and riboflavin metabolism. Amino acid metabolism and fat metabolism were the prominent pathways in both PWNs treated with NSPmBx03 and fungus PWNs. The results indicated that the endobacterium and other bacteria could affect detoxification-related metabolism. The bacteria of fungus PWNs affected gene expression to a greater extent than *S. maltophilia* NSPmBx03. In addition, endobacteria and other bacteria may help nutrition metabolism of PWNs.

### 2.8. Validation of DEGs by Real-Time Quantitative PCR

**Figure 6:**
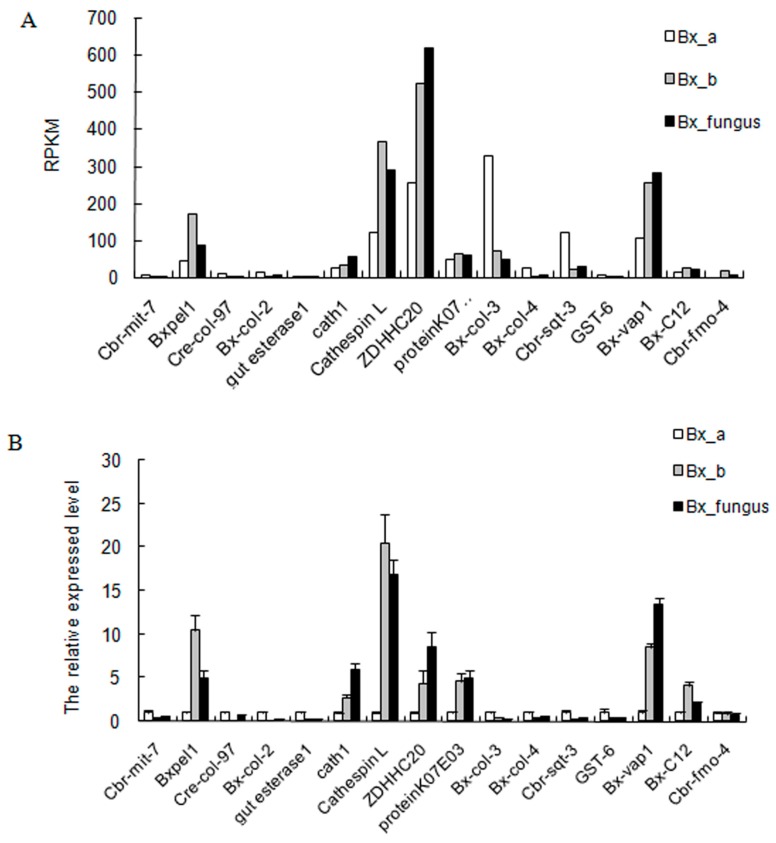
Relative gene expression. (**A**) Expression levels of 16 genes in sequencing results. RPKM, reads per kilobase per million reads; (**B**) qRT-PCR results of 16 differentially expressed genes under sterile, endobacterium treatment, and fungus conditions. White bars indicate expression levels of the genes in control samples (Bx_a); gray bars indicate expression levels of genes in endobacterium-treated PWN samples (Bx_b); black bars indicate expression levels of genes in fungus PWN samples (Bx_fungus). Data represent the means ± SD (*n* = 3). SD, standard deviations.

To validate the transcriptome results, 16 genes were selected at random from the DEGs and analyzed with qRT-PCR. We designed specific primers for seven up-regulated genes (*Bxpel2*, *cath 1*, *cathespin L*, *ZDHHC20*, *protein KO7*, *Bx-vap1*, and *Bx-C12*) and nine down-regulated genes (*Cbr-mit-7*, *Cre-col-97*, *Bx-col-2*, *gut esterase1*, *Bx-col-3*, *Bx-col-4*, *Cbr-sqt-3*, *GST-6*, and *Cbr-fmo-4*). The expression tendency of the 16 candidate genes for qRT-PCR was similar to expression tendency observed by RNA-seq, demonstrating reliability of transcriptome data (Figure 6).

### 2.9. Symptoms of Pines Inoculated with PWNs

**Figure 7:**
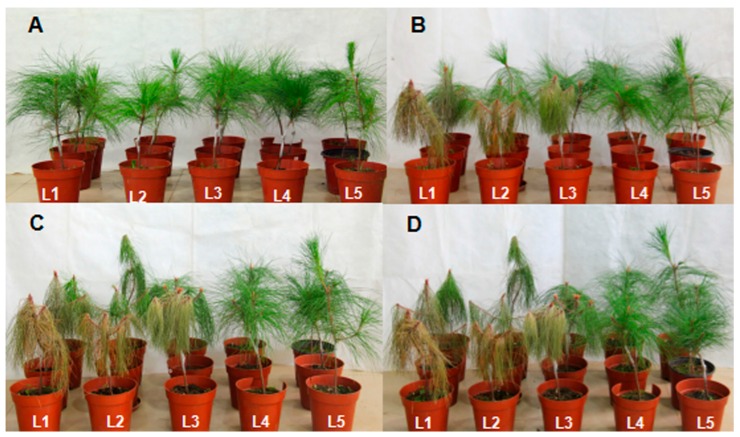
Symptoms of *P. massoniana* inoculated with different PWN treatments. (**A**) Symptoms after 0 day of inoculation; (**B**) Symptoms after 21 days; (**C**) Symptoms after 26 days; (**D**) Symptoms after 30 days. Pines inoculated with: L1, fungus *B. xylophilus*; L2, *B. xylophilus* treated with *S. maltophilia* NS PmBx 03; L3, aseptic *B. xylophilus*; L4, *S. maltophilia* NSPmBx 03; and L5, sterile water.

Pines inoculated with different PWNs showed differences in symptoms. The time of initial symptoms of fungus PWNs and PWNs treated with bacteria appeared 13 days after inoculation and 17 days for aseptic PWNs. After 21 days of inoculation, pines inoculated with fungus PWNs had an infection rate of 100% and a disease severity index (DSI) of 83.3, while *S. maltophilia* NSPmBx03 treatment infection rate was 66.7% and DSI was 58.3; aseptic PWN treatment infection rate was 33.3% and DSI was 33.3 (Table 1). After 26 days of inoculation, infection rates of all pines with different treated PWNs were 100%, but aseptic PWNs had the lowest DSI. After 30 days of inoculation, only one pine inoculated with aseptic PWNs was not completely wilted. After 35 days of inoculation, all pine needles were brown and wilting. Pines inoculated with *S. maltophilia* NSPmBx03 and sterile water remained healthy (Figure 7). These results indicated that the *S. maltophilia* NSPmBx03 enhanced the virulence of PWNs.

## 3. Discussion

Symbiotic relationships between nematodes and bacteria are common in nature. Recent studies showed that bacteria carried by the PWN were likely to play a role in PWD [18,21,28]. Zhang *et al.* [29] reported that *Stenotrophomonas* was the most dominant group in the PWN. Wu *et al.* [13] isolated several endobacterial strains from PWNs, and *S. maltophilia* was a dominant species. The specific and functional diversities of endobacteria from various isolates of *B. xylophilus* have been studied [13]. Tian *et al.* [21] reported that endobacteria *S. maltophilia* NSBx.14 could decrease the reproduction of ZL1 in fungus-conditions. However, the molecular mechanisms between endobacteria and the PWN are poorly understood. In this study, we sequenced the transcriptome of *B. xylophilus* treated with *S. maltophilia* NSPmBx03 for the first time and found that NSPmBx03 affected the gene expression level of *B. xylophilus* (Table S3).

Pectinases are the primary cell-degrading enzymes of the PWN secretome, intrinsically related to nematode pathogenicity in host pines [30,31]. Pectin is the most complex component of the pine cell wall polysaccharides, and plays a critical role in resisting invasion by PWN [32]. To invade the host successfully, PWN needs to break down this barrier. Therefore, pectinases are essential to allow PWN to infect its plant host. Qiu *et al.* found that pectin lyase genes of PWNs were highly expressed when PWNs infected *P. thunbergii* [33]. From the secretion of the PWN esophageal gland, DeBoer *et al.* [34] cloned *Bxpel1* and *Bxpel2*, which enhanced the ability of PWNs to feed and migrate in the host pine. Our results showed that the endobacterium *S. maltophilia* NSPmBx03 affected expression of genes of carbohydrate active enzymes in the PWN. The expression of the pectate lyase gene *BUX.s01530.1* was up-regulated 5.5-fold in fungus PWNs and 5-fold in PWN treated with endobacteria compared to control treatment (Figure 2). After *P. massoniana* was inoculated with aseptic PWNs, PWNs treated with endobacterium, and fungus PWNs, respectively, we found that the fastest group lead to pine trees wilting was fungus PWNs, followed by PWNs treated with endobacterium, and the last was the pines inoculated with aseptic PWNs. As previously reported, the bacteria species associated with *B. xylophilus* AmA3 were *Stenotrophomonas*, Rhizobiaceae (unclassified), *Chitinophaga*, Oxalobacteraceae (unclassified), and *Ochrobactrum* [23]. These results indicated that *S. maltophilia* NSPmBx03 and some other bacteria associated with the PWN could affect pectate lyase gene expression, which may play an important role in parasitizing pines.

Phytotoxins such as benzoic acid, 8-hydroxycarbotanacetone, and 10-hydroxyverbenone were identified and characterized from PWN-infested pines [35]. These toxic substances could defend against invasion and decrease the reproduction and migration rate of the PWN in host pines [36]. Futai *et al.* [37] found that tannic acid significantly accumulated in parenchymal cells of *P. thunbergii* after PWN invasion. Additionally, PWN inhabits the resin canals of host pines [38] and the resin is a complex mixture of compounds, including terpenoids and cyclic aromatic compounds [39]. These compounds are likely to have nematocidal activity. PWNs must detoxify these toxic compounds in order to invade the pines successfully. Our results showed that abundant derived enzymes were assigned to xenobiotic metabolism pathways in the transcriptome of PWNs treated with NSPmBx03 and fungus PWN. The major pathways were drug metabolism *CYP*, metabolism of xenobiotics by *CYP* and glutathione metabolism (Figure 5). *CYP* and *GST* were the primary DEGs of detoxification (Figure 3). These results were consistent with those of Yan *et al.*, who reported that *CYP* and *GST* pathways were the primary detoxification metabolic pathways in PWN [40]. The detoxification process of PWN has been divided into three successive phases [27]. First, *CYP*s are the most important group of the first phase proteins, making molecules more suitable substrates for downstream. Second, the actual detoxification reaction occurs with *GST*s and UDP-glucuronosyl transferases (UGTs) as essential enzymes. In the third phase, an *ABC* transporter is responsible for the efflux of detoxification molecules. Vicente *et al.* [41] reported that some PWN-associated bacteria could increase nematode survival under strong oxidative stress. Xu *et al.* [42] identified the function of *CYP* genes (*BxCYP33C9*, *BxCYP33C4*, and *BxCYP3D3*) in *B. xylophilus*, and revealed that these genes may affect their viability, proliferation, pesticide metabolism and pathogenicity. In our study, a number of detoxification-related genes, including *GST*, *ABC* transporter, venom allergen-like protein VAP1, retinol dehydrogenase, and *CYP*, were greatly up-regulated in the PWNs treated with *S. maltophilia* NSPmBx03 (Figure 3). These results indicated that NSPmBx03 played an important role in the xenobiotic detoxification of PWN, which may help PWN cope with a difficult environment.

A recent study showed that *Pseudomonas* spp. could promote the reproduction of *B. xylophilus* [43]. However, some other bacteria, such as *Pantoea* sp., *Serratia marcescens*, *Buttiauxella agrestis* and *Enterobacter amnigenus* had an inhibitory effect [43]. Tian *et al.* [21] reported that endobacteria isolated from highly virulent PWN could partially promote the development of nematodes, while endobacteria isolated from lowly virulent PWN may retard the growth of nematodes. Additionally, *S. maltophilia* NSBx.14, obtained from the highly virulent ZL1 strain of *B. xylophilus*, can prolong the development time of nematode embryos [22]. We found that the expression of juvenile and embryo development-related genes in PWNs treated with *S. maltophilia* NSPmBx03 and fungus PWNs were significantly down-regulated (Figure 4). As in previous results, we found that aseptic PWN had a higher reproduction rate than PWN treated with bacteria on *B. cinerea*, but completely opposite results were obtained for *P. massoniana* [44]. Cheng *et al.* [45] reported that *Stenotrophomonas* was the most common genera of symbiotic bacteria with PWN, and may play an important role in regulating ecological function. Qiu *et al.* [33] found that the expression levels of the pectate lyase, *CYP*, UGT, and *ABC* transporter genes, and reproduction-related genes of PWN associated with bacteria were up-regulated dramatically by growth on *P. thunbergii* compared with growth on *B. cinerea*. Therefore, we speculated that the effect of bacteria on PWN differed in fungus-conditions and parasitic conditions. In fungus-conditions, bacteria might be detrimental to nematodes to some extent. In parasitic conditions, the high-level expression of cell wall degradation-related and detoxification-related genes could help the PWN successfully parasitize pines and grow better in the host. These results will help us to perceive the underlying nature of the relationship between pine wood nematode and bacteria. However, the role of endobacteria in PWD requires further experimental verification.

## 4. Materials and Methods

### 4.1. The PWN and Its Endobacteria

PWNs (AmA3) were isolated from infected *P. massoniana* in Maanshan, Anhui, China [46]. An endobacterium *S. maltophilia* NSPmBx03 was initially isolated from inside the body of PWNs extracted from infected *P. massoniana* in Nanjing, Jiangsu, China. This bacterium was frequently isolated from PWN strains in our previous study [13]. The strains of the PWN and the endobacterium were provided by the Jiangsu Key Laboratory for Prevention and Management of Invasive Species.

### 4.2. Preparation of Bacteria-Free PWNs and Treatment of Aseptic PWNs with Endobacterium

A suspension of 6000 PWNs was used to inoculate six-year-old *P. massoniana* in the field (Nanjing Forestry University, Nanjing, China) [33]. The PWNs were isolated from dead *P. massoniana* using a Baermann funnel under aseptic conditions. Then, the PWNs were pipetted into a potato dextrose agar plate (6-cm diameter) containing *B. cinerea*. After growth at 25 °C for 7 days, the nematodes were collected in 10-mL centrifuge tubes and treated as fungus PWN (Bx_fungus).

The above-mentioned suspension (1 mL) of PWNs was poured onto a sterilized cover slip in a Petri dish and incubated at 25 °C for depositing eggs during 4–6 h, and the nematodes discarded after egg deposition [47]. The nematode eggs were collected and soaked in 15% H_2_O_2_ for 60 min at 25 °C. The eggs were rinsed three times with sterile water and placed on mycelia of *B. cinerea* grown on PDA medium at 25 °C in darkness. The hatched nematodes were collected in 10-mL centrifuge tubes under aseptic conditions and treated as aseptic PWNs (Bx_a). Aseptic PWNs was the control treatment.

The endobacterial strain of *S. maltophilia* NSPmBx03 maintained on slant nutrient agar medium in tubes was transferred to 50 mL of beef extract-peptone liquid medium and cultured on a shaker for 24 h. The concentration of *S. maltophilia* NSPmBx03 was adjusted to 8.0 × 10^8^ CFU (Colony-Forming Units) and 200 µL of suspension was sprayed onto PDA plates containing *B. cinerea* [21]. Each plate was inoculated with 1000 bacteria-free nematodes (AmA3) and incubated for 7 days at 25 °C. Then PWNs were collected into 10-mL centrifuge tubes with a Baermann funnel under aseptic conditions. The nematodes (Bx_b) were washed three times with sterile water.

### 4.3. RNA Extraction, cDNA Preparation and Illumina Sequencing

Nine samples (each treatment had three replicates) were immediately frozen in liquid nitrogen and stored at −75 °C until use. Total RNA was respectively extracted from frozen nematode samples (Bx_a, Bx_b, and Bx_fungus), using a RNAprep Kit (Tiangen, Beijing, China) and purified using a RNAclean Kit (Tiangen) according to the manufacturer’s instructions. RNA integrity and quantity were determined with an Agilent 2100 Bioanalyzer (Agilent Technologies, Redwood City, CA, USA) before cDNA synthesis. One of the three RNA samples from each treatment PWN was used for RNA-seq. The mRNA was isolated from total RNA by Oligo (dT) magnetic beads. Isolated mRNA strands were fragmented and used as templates to synthesize cDNA using a cDNA Synthesis SystemKit (Roche Applied Science, Mannheim, Germany) [48]. The short fragments that were purified and resolved with EB buffer were subjected to end repair and addition of single nucleotide A (adenine). After that, the short fragments were connected with adapters. The qualified fragments of three cDNA samples were respectively selected for PCR amplification as templates. The quality of cDNA libraries was determined with an Agilent 2100 Bioanalyzer and ABI StepOnePlus Real-Time PCR System (Life Technologies, Grand Island, NY, USA). Each cDNA library was sequenced in a single lane of the Illumina HiSeq^TM^ 2000 system using paired-end protocols according to the manufacturer’s instructions at BeiJing Genomics Institute (Shenzhen, China). The RNA-Seq experiments have three technical replicates. The raw data were deposited in the NCBI Short Read Archive (SAR) and are accessible through SAR accession numbers: SRR2336946, SRR2342509, and SRR2342510.

To obtain clean reads, adapter, reads in which unknown bases were more than 10%, low quality reads were removed from raw reads using the SeqPrep program (https://github.com/jstjohn/SeqPrep).

### 4.4. Read Mapping and DEG Analysis

The clean reads of three PWN samples with different treatment methods were mapped to the genome of *B. xylophilus* (http://www.ncbi.nlm.nih.gov/genome) using SOAP aligner/SOAP2 [49], allowing for maximum mismatch of 5 bp. Gene expression levels of three PWN samples were calculated by reads per kilobase transcriptome per million mapped reads (RPKM) method [50]. DEGs were defined as those with changes of at least two-fold between samples (Bx_a *vs.* Bx_b, and Bx_a *vs.* Bx_fungus) and at FDR ≤ 0.001 [51,52], excluding genes where the RPKM was 0 in either sample. Cluster analysis of gene expression patterns was conducted with cluster [53] and Java Treeview [54] software.

Finally, the DEGs were analyzed for enrichment of GO and KEGG pathway terms. All DEGs were mapped to GO terms in the database (http://www.geneontology.org). Then, gene numbers in every term were calculated, and hypergeometric test was used to detect the notably enriched GO terms [55]. The calculated *p*-value was subject to Bonferroni correction [56]. Set the corrected *p*-value ≤0.05 as a threshold. GO terms meeting this threshold were selected as notably enriched in DEGs. KEGG (www.genome.jp/kegg/) was used to perform metabolic pathways or signal transduction pathways enrichment analysis of DEGs. Significantly enriched pathways were identified in DEGs by comparing with the whole genome background. The method of calculation was used as that in GO analysis.

### 4.5. qRT-PCR Validation

To verify the RNA-seq results, nine extracted RNA samples of different treatment PWNs were used for qRT-PCR analysis. The first-strand cDNA was synthesized with Prime Script 1st strand cDNA synthesis Kit (TaKaRa, Shiga, Japan), and the cDNA samples were diluted to 20 ng/µL. Sixteen gene-specific primers (Table S2) were designed using Primer Premier 5.0 software. The *Actin* gene of *B.xylophilus* was used as internal control: *Actin* forward primer 5′-GCAACACGGAGTTCGTTGTA-3′ and reverse primer 5′-GTATCGTCACCAACTGGGAT-3′. The qRT-PCR was performed using the ABI 7500 system (Applied Biosystems, Waltham, MA, USA), with a 20-µL final reaction volume, which contained 2 µL of template, 10 µL of SYBR Premix Ex Tap, 0.4 µL of ROX Reference Dye II, 0.4 µL of forward primer, 0.4 µL of reverse primer, and 6.8 µL of ddH_2_O. The PCR amplification was performed as follows: 40 cycles of 95 °C for 30 s, and 60 °C for 34 s. The relative expressed levels in selected genes of three PWN samples were evaluated using the relative quantification method (2^−ΔΔ*C*t^) [57]. These experiments were biological (*n* = 3) and technical (*n* = 3) repeated.

### 4.6. Observation of P. massoniana Symptoms for Different Treatments of PWNs

The experiment was conducted in a greenhouse. All *P. massoniana* (2 years old) were disinfected by spraying with 75% ethyl alcohol. After that, 0.5 mL of suspension (3000 nematodes) of different treatments of PWNs (Bx_a, Bx_b, and Bx_fungus) were respectively pipetted into cutting wounds (0.5 cm in length) in *P. massoniana* seedlings at about 20 cm above the soil level. Sterile water was used as control. The wounds on *P. massoniana* were sealed by Parafilm. Each treatment contained three replicates. The inoculated seedlings were placed in the greenhouse. PWD symptoms were evaluated and categorized as 0–4 [58]. The categories were as follows: 0 = all needles were green; 1 = 0%–25% needles discolored and turned yellow; 2 = 25%–50% needles turned yellow; 3 = 50%–75% needles turned yellow; and 4 = 75%–100% needles turned yellow. The infection rates and DSI were calculated with the formulae as follow:

$$\text{Infection rates}=\frac{\sum \text{Number of infected plants}}{\text{Total number of plants}}\times 100\%$$

$$\text{DSI}=\frac{\sum \text{Number of disease plants}\times \text{symptom stage}}{\text{Total number of plant}\times \text{highest symptom stage}}\times 100$$

## 5. Conclusions

The results demonstrated that the endobacterium *S. maltophilia* NSPmBx03 could affect expression levels of genes related to cell wall degradation, detoxification, and reproduction genes in PWNs and enhance the virulence of nematodes. This suggests that *S. maltophilia* NSPmBx03 played an important role in the gene expression of PWN. These results provide insights into the regulatory mechanisms involved in the interaction between nematodes and bacteria, which will facilitate a better understanding of the role of bacteria in PWD.

## Figures and Tables

**Table 1:** Symptoms of *P. massoniana* caused by aseptic *B. xylophilus*, *B. xylophilus* treated with *S. maltophilia* NSPmBx03, and fungus *B. xylophilus*. Bx_a: aseptic *B. xylophilus*, Bx_b: *B. xylophilus* treated with *S. maltophilia* NSPmBx03, Bx_fungus: fungus *B. xylophilus.*

Time of Inoculation (d)	Bx_a		Bx_b		Bx_fungus	
	Infection Rate (%)	Disease Severity Index	Infection Rate (%)	Disease Severity Index	Infection Rate (%)	Disease Severity Index
13	0	0	33.3	25	66.7	50
21	33.3	33.3	66.7	58.3	100	83.3
26	100	83.3	100	91.7	100	100
30	100	91. 7	100	100	100	100

## Supplementary Materials

Supplementary materials can be found at http://www.mdpi.com/1422-0067/17/6/778/s1.
