# Supplementary material for: Effects of Endobacterium (Stenotrophomonas maltophilia) on Pathogenesis-Related Gene Expression of Pine Wood Nematode (Bursaphelenchus xylophilus) and Pine Wilt Disease
Source: Int J Mol Sci. 2016 May 25;17(6):778. doi: 10.3390/ijms17060778 (PMC4926329; doi:10.3390/ijms17060778)
Supplement: Supplementary file 1 [file ijms-17-00778-s001.pdf]

Supplementary Materials: Effects of Endobacterium (*Stenotrophomonas maltophilia*) on Pathogenesis-Related Gene Expression of Pine Wood Nematode (*Bursaphelenchus xylophilus*) and Pine Wilt Disease

Long-Xi He, Xiao-Qin Wu, Qi Xue and Xiu-Wen Qiu

Table S1. The results of sequences of differently treated *B. xylophilus* mapped to the reference genome.

Map to Genome (Genome/Gene)	*B. xylophilus* with Different Treatment		
	Bx_a	Bx_b	Bx_Fungus
Total reads	47,931,208	48,015,322	48,031,606
Total basepairs	4,313,808,720	4,321,378,980	4,322,844,540
Toal mapped reads (percentage)	75.43%/71.58%	75.06%/72.66%	75.04%/71.98%
Perfect match (percentage)	54.33%/52.65%	54.2%/53.78%	54.48%/53.51%
Mismatch ≤ 5 bp (percentage)	21.11%/18.93%	20.86%/18.88%	20.56%/18.47%
Unique match (percentage)	74.02%/70.78%	73.78%/71.88%	73.79%/71.26%
Multi-position match (percentage)	1.41%/0.8%	1.27%1/0.78%	1.25%/0.72%
Total unmapped reads (percentage)	24.57%/28.42%	24.94%/27.34%	24.96%/28.02%

Table S2. Primer sequences of differentially expressed genes in *B. xylophilus* for qRT-PCR analysis.

Gene ID	Gene Name	Function	Primer Sequence (5′ to 3′)
BUX.s01109.547	*Cbr-mit-7*	Thyroid peroxidase	F: AACAAATGAATCCCTCCTG<br>R: GTGACCGAAACGATAGGC
BUX.s01661.75	*Bxpel1*	Pectate lyase	F: AAGAGCGGGCAGACCTAC<br>R: GGATTTCCCAGGACAACAT
BUX.s00364.216	*Cre-col-97*	Collagen protein	F: AAACAACTACCCACCATCCTT<br>R: TTCCTTGGCGTCCATCTT
BUX.s00055.267	*Bx-col-2*	CBN-col-68 protein	F: TTCTGCGTTCACAGATCCAA<br>R: CGGCGTCATCTCCGTAAGT
BUX.s00713.1002	*gut esterase1*	Carboxylesterase	F: CTCAAATGGGTCTATGACA<br>R: GGCTTAGAGTAGGGAGAAA
BUX.s00813.53	*cath1*	Cathepsin F	F: GTAATCATCGCTGTAATCTGT<br>R: ATTCGTTCAAGGGTTTCT
BUX.s00813.52	*Cathespin L*	Cathespin L	F: ATGTAATGGCGGATGGTC<br>R: TGGATTTGCAGCTAGGAA
BUX.s01254.165	*ZDHHC20*	ZDHHC20 Protein	F: GAATAGTCCGAGACTCAAAT<br>R: TCGTTATCTTCCTCGTCA
BUX.s00116.359	*Protein K07E3*	Catalytic activity	F: GGACCGACCTTTGGGATT<br>R: CGGCAGCGAGGAGATTGT
BUX.s00532.5	*Bx-col-3*	Juvenile development	F: ATTACGAGAACCGCATCAAAGC<br>R: TGGGCAGCGTGAAGCAGA
BUX.s00116.877	*Bx-col-4*	Juvenile development	F: CACTCGGTTCGTCGGCAAAT<br>R: ACGGGCGGTTCTGTTGGA
BUX.s01518.49	*Cbr-sqt-3*	Embryo development	F: AGTCCGATGGCCGTATGAA<br>R: TGACCTGGCTGACGTAGTTGTA
BUX.s01254.333	*GST-6*	Glutathione *S*-transferase activity	F: CCCATCTACAACAACCTCC<br>R: CAAGTATTCGCTGACCACA
BUX.s00579.85	*Bx-vap1*	Venom allergen-like protein VAP1	F: GCCCATAACAACCTTCGG<br>R: CATTCGTCGGCTTTGGAT
BUX.s00397.15	*Bx-C12*	Beta-1,4-endoglucanase activity	F: ACGGATTTGCTGCTGTGA<br>R: CAGGTAGCCGACTCCACA
BUX.s00713.800	*Cbr-fmo-4*	Monooxygenase activity	F: GACTACAACAACGGATGG<br>R: TTGGCTTCAAACGACTCTAC

F, forward primer; R, reverse primer.

Table S3. The high differently expressed genes in *B. xylophilus* treated with endobacterium and wild-type *B. xylophilus.* Bx_b: *B. xylophilus* treated with endobacterium, Bx_w: fungus *B. xylophilus.* '-' represent down-regulated.

Gene ID	Annotation	Change Fold in Bx_b (Fold)	Change Fold in Bx_Fungus (Fold)
BUX.s01147.112		−10.47	−4.08
BUX.s00240.28		−9.02	−2.01
BUX.s00364.216	Hypothetical protein CRE_26523	−8.84	−3.04
BUX.s00240.41		−7.31	−6.41
BUX.s00116.877		−6.50	−3.71
BUX.s00116.209		−6.07	−3.57
BUX.s01143.351	*C. briggsae* CBR-GRL-9 protein	−5.98	−2.69
BUX.s00351.83		−5.75	−4.93
BUX.s00055.267	CBN-COL-68 protein	−5.71	−2.70
BUX.s01145.106	C. briggsae CBR-COL-156 protein	−5.58	−3.13
BUX.s01518.47	Hypothetical protein CRE_05777	−5.49	−2.57
BUX.s00813.30	Hypothetical protein WUBG_07659	−5.36	−2.31
BUX.s00358.44		−5.34	−4.23
BUX.s01661.24	I-Branching enzyme family protein	−5.33	−3.66
BUX.s01518.49	Hypothetical protein CAEBREN_32352	−5.26	−4.43
BUX.s01254.267	Protein COL-65	−5.24	−8.82
BUX.s01147.16		−5.21	−3.00
BUX.s01653.305	Nematode cuticle collagen N-terminal domain containing protein	−5.16	−4.15
BUX.s01109.625	Hypothetical protein CAEBREN_25275	−5.14	−3.96
BUX.s01653.308	C. briggsae CBR-COL-156 protein	−5.09	−3.49
BUX.s01259.16	Hypothetical protein CRE_11135	−5.00	−2.31
BUX.s01513.216	Hypothetical protein CBG20858	−5.00	−2.30
BUX.s00351.199	Protein H10E21.4	−4.96	−3.26
BUX.s01254.196	Hypothetical protein CRE_01635	−4.95	−3.27
BUX.s00116.244		−4.94	−2.51
BUX.s01142.4	CRE-COL-130 protein	−4.93	−5.65
BUX.s00713.745		−4.93	−4.21
BUX.s00100.1		−4.81	−2.54
BUX.s00422.105	Hypothetical protein CAEBREN_15197	−4.77	−2.06
BUX.s00609.14	*C. briggsae* CBR-COL-123 protein	−4.71	−3.14
BUX.s01063.14		−4.67	−3.25
BUX.s01653.307	*C. briggsae* CBR-COL-65 protein	−4.59	−3.47
BUX.s00252.138	Hypothetical protein LOAG_00897	−4.53	−5.00
BUX.s00298.1		−4.52	−5.44
BUX.s00747.2	Hypothetical protein Bm1_36710	−4.47	−4.32
BUX.s01109.128	Cuticle collagen 13	−4.45	−2.91
BUX.s00532.5	Cuticle preprocollagen	−4.45	−6.86
BUX.s01518.48	Hypothetical protein CAEBREN_32352	−4.26	−5.37
BUX.s00713.945	Cuticular collagen	−4.25	−2.91
BUX.s00298.56	Hypothetical protein CAEBREN_10142	−4.21	−2.81
BUX.s00460.301	Cuticle collagen 13 precursor	−4.21	−5.41
BUX.s00119.27	*C. briggsae* CBR-NHX-3 protein	−4.20	−2.72
BUX.s00713.946	Collagen protein	−4.17	−2.52
BUX.s00713.944	Col-180-1	−4.16	−3.63
BUX.s01144.229		−4.14	−5.73
BUX.s01092.240		−4.13	−3.54
BUX.s00579.123		−4.12	−3.00
BUX.s00358.45		−4.11	−3.89
BUX.s00252.94	Hypothetical protein LOAG_17054	−4.10	−3.72
BUX.s00647.64		−4.08	−3.66
BUX.s01429.2		−3.98	−3.91
BUX.s00460.148	DB module family protein	−3.98	−2.40
BUX.s01254.268	CRE-ROL-8 protein	−3.95	−2.78
BUX.s00713.1056		−3.93	−5.58
BUX.s01092.72	Cathepsin B-like cysteine protease 2	−3.93	−3.41
BUX.s00116.36	Hypothetical protein CBG09274	−3.93	−2.74
BUX.s01144.113	Hypothetical protein LOAG_18235	−3.88	−3.29

Table S3. *Cont.*

Gene ID	Annotation	Change Fold in Bx_b (Fold)	Change Fold in Bx_Fungus (Fold)
BUX.s01187.19	Hypothetical protein Bm1_50485	−3.87	−2.19
BUX.s01142.58		−3.85	−4.47
BUX.s00333.136	C. briggsae CBR-LON-3 protein	−3.80	−3.65
BUX.s00240.3	Protein LON-3	−3.79	−3.82
BUX.s01254.121	Nematode cuticle collagen N-terminal domain containing protein	−3.78	−4.98
BUX.s01143.148	Hypothetical protein CBG23119	−3.78	−3.25
BUX.s00110.82	Hypothetical protein CBG14246	−3.78	−2.52
BUX.s00055.304	Hypothetical protein LOAG_01570	−3.77	−2.85
BUX.s00532.4	Putative COLlagen	−3.74	−6.05
BUX.s00713.39	Protein C25E10.12	−3.73	−3.77
BUX.s00139.133	C. briggsae CBR-COL-40 protein	−3.71	−3.22
BUX.s01142.5	collagen protein	−3.69	−5.10
BUX.s00036.123	Hypothetical protein Bm1_38265	−3.67	−6.00
BUX.s00713.1009	Hypothetical protein KGM_03271	−3.60	−2.66
BUX.s00116.149	Ground-like domain containing protein	−3.60	−3.55
BUX.s00364.224	COL5A1 protein	−3.59	−6.91
BUX.s00600.66	Hypothetical protein LOAG_08929	−3.58	−3.92
BUX.s01653.296	C. briggsae CBR-COL-107 protein	−3.57	−6.55
BUX.s01109.130	Protein COL-160	−3.56	−5.32
BUX.s00351.445	Protein ELO-4	−3.48	−2.85
BUX.s00647.95	Hypothetical protein CRE_17077	−3.48	−5.15
BUX.s01109.332	Hypothetical protein LOAG_04512	−3.47	−4.08
BUX.s01063.43	CRE-TAG-297 protein	−3.47	−2.34
BUX.s01038.94	Hypothetical protein CAEBREN_16721	−3.43	−2.14
BUX.s00460.489	Hypothetical protein CRE_20821	−3.43	−3.90
BUX.s00713.1051		−3.41	−2.29
BUX.s01438.96		−3.41	−2.16
BUX.s01143.316		−3.40	−2.32
BUX.c00697.1		−3.39	−3.29
BUX.s01145.76	Hypothetical protein CAEBREN_06992	−3.39	−3.75
BUX.s01063.44	C. briggsae CBR-TAG-297 protein	−3.39	−3.46
BUX.s00579.3		−3.39	−2.20
BUX.s00036.151	CRE-COL-45 protein	−3.39	−3.12
BUX.s01142.59		−3.36	−5.00
BUX.s00713.40	Protein WRT-8	−3.35	−2.22
BUX.s01109.547	Protein MLT-7	−3.34	−2.44
BUX.s00119.37	Hypothetical protein LOAG_04049	−3.34	−2.39
BUX.s00964.26	Hypothetical protein LOAG_05885	−3.34	−2.52
BUX.s00961.57		−3.31	−2.93
BUX.s01109.563	Nematode cuticle collagen N-terminal domain containing protein	−3.28	−3.21
BUX.s01092.247		−3.27	−2.78
BUX.s01109.45	Cuticlin 1	−3.27	−2.45
BUX.s01187.11	Hypothetical protein CAEBREN_00254	−3.26	−2.34
BUX.s00351.186	Hypothetical protein LOAG_06354	−3.25	−3.50
BUX.s00725.36	Hypothetical protein WUBG_10966	−3.25	−3.94
BUX.s01144.96	Nematode cuticle collagen N-terminal domain containing protein	−3.24	−3.55
BUX.s00713.1123	Hypothetical protein CAEBREN_14564	−3.24	−2.58
BUX.s00351.158	C. briggsae CBR-PQN-46 protein	−3.21	−2.24
BUX.s00055.287	PAN domain-containing protein	−3.20	−4.07
BUX.s00119.8	CBN-CUT-1 protein	−3.20	−2.30
BUX.s00713.838	Hypothetical protein DAPPUDRAFT_302039	−3.19	−2.72
BUX.s00813.67	Protein BUS-12, isoform a	−3.17	−4.35
BUX.s01178.2	CRE-ELO-4 protein	−3.17	−2.70
BUX.s00649.21	Hypothetical protein Bm1_38120	−3.15	−4.32
BUX.s00422.286	Lipase family protein	−3.15	−2.74
BUX.s00422.163	Hypothetical protein LOAG_05181	−3.14	−2.94

Table S3. *Cont.*

Gene ID	Annotation	Change Fold in Bx_b (Fold)	Change Fold in Bx_Fungus (Fold)
BUX.s00460.502	CBN-WRT-4 protein	−3.13	−3.61
BUX.s00364.94	Protein DPY-2	−3.13	−5.12
BUX.s01254.333	Protein GST-6	−3.13	−3.34
BUX.s00139.89	Hypothetical protein BRAFLDRAFT_59053	−3.11	−11.95
BUX.s00713.779	CRE-MLT-11 protein	−3.10	−2.62
BUX.s00351.118	CBR-CBN-1 protein	−3.07	−3.76
BUX.s00713.209	Collagen col-34	−3.06	−2.50
BUX.s01281.492		−3.06	−2.48
BUX.s00351.297	Hypothetical protein LOAG_04701	−3.06	−2.34
BUX.s00333.141	Cuticlin 1, partial	−3.05	−2.58
BUX.s00543.37	C. briggsae CBR-DPY-8 protein	−3.05	−2.85
BUX.s00351.228	Protein LET-805, isoform b	−3.04	−2.99
BUX.s00649.20	Protein LPR-6, isoform a	−3.01	−2.91
BUX.s01063.215	Protein PTR-18	−3.01	−2.60
BUX.s01662.136	CRE-SQT-2 protein	−3.00	−4.13
BUX.s00351.227	Hypothetical protein H19M22.2—Caenorhabditis elegans	−2.99	−2.46
BUX.s01494.1	Protein B0365.9	−2.98	−5.63
BUX.s00460.374	WRT-10 protein	−2.98	−5.78
BUX.c00436.1		−2.97	−3.96
BUX.s01661.37	Protein COL-46	−2.97	−3.95
BUX.s01147.26		−2.95	−2.95
BUX.s00813.116	LPR-4 protein	−2.95	−3.87
BUX.s00364.221	Collagen protein	−2.95	−5.45
BUX.s00055.338	Hypothetical protein Bm1_38110	−2.94	−3.25
BUX.s01144.205		−2.94	−3.63
BUX.s00364.218	COL5A1 protein	−2.93	−2.14
BUX.s00298.242	Hypothetical protein CAEBREN_13878	−2.93	−2.48
BUX.s00116.855		−2.89	−3.49
BUX.s01109.545	Hypothetical protein CAEBREN_06068	−2.88	−2.80
BUX.s01109.461		−2.88	−2.16
BUX.s00983.1	Hypothetical protein LOAG_17799	−2.86	−4.11
BUX.s01653.440		−2.85	−2.66
BUX.s00713.673		−2.85	−2.38
BUX.s00116.713	CRE-PTR-4 protein	−2.83	−2.64
BUX.s01281.367	Period, partial	−2.82	−3.26
BUX.s00364.92	Hypothetical protein LOAG_01555	−2.82	−3.42
BUX.s01254.264	Cuticular collagen	−2.80	−2.68
BUX.s01187.18	Hypothetical protein WUBG_01354	−2.79	−2.04
BUX.s00460.143	Hypothetical protein CRE_22550	−2.79	−2.20
BUX.s01226.79	Hypothetical protein LOAG_17054	−2.79	−3.67
BUX.s01653.325	Hypothetical protein WUBG_06137	−2.78	−2.25
BUX.s00116.812	Hypothetical protein Bm1_28255	−2.77	−3.35
BUX.s00351.374	Hypothetical protein LOAG_02391	−2.76	−3.80
BUX.s01653.301	Hypothetical protein CAEBREN_14780	−2.75	−2.89
BUX.s00713.6	2.96412e-66/Protein R57.2	−2.74	−2.22
BUX.s00110.32	3-oxoacyl-[acyl-carrier-protein] reductase	−2.74	−7.96
BUX.s01150.21	CBN-DAO-2 protein	−2.74	−2.77
BUX.s01143.56	Hypothetical protein CAEBREN_30296	−2.74	−2.67
BUX.s00961.65	Protein F29G6.1	−2.73	−2.39
BUX.s00351.368	Hypothetical protein Bm1_05905	−2.72	−4.25
BUX.s00422.593	Protein LIT-1, isoform d	−2.72	−2.74
BUX.s00119.69		−2.72	−4.33
BUX.s00422.548	Angiotensin-converting enzyme family protein	−2.72	−2.74
BUX.s00579.202	3-oxoacyl-[acyl-carrier-protein] reductase	−2.72	−7.73
BUX.s01109.245	Hint module family protein	−2.70	−2.48
BUX.s01259.35	Macrophage migration inhibitory factor	−2.70	−6.86
BUX.s00713.800	*C. briggsae* CBR-FMO-4 protein	−2.69	−6.36

Table S3. *Cont.*

Gene ID	Annotation	Change Fold in Bx_b (Fold)	Change Fold in Bx_Fungus (Fold)
BUX.s00117.41		−2.68	−2.07
BUX.s01653.160	PAN domain-containing protein	−2.67	−3.78
BUX.s01226.46	Hypothetical protein CAEBREN_15384	−2.67	−9.72
BUX.s01653.324		−2.67	−5.70
BUX.s00116.625	CBN-CLEC-180 protein	−2.67	−3.17
BUX.s01438.105	Hypothetical protein CAEBREN_19046	−2.66	−2.84
BUX.s00579.725	Protein BUS-8	−2.66	−2.59
BUX.s00088.1	*C. briggsae* CBR-COL-88 protein	−2.65	−2.20
BUX.s00333.161	Hypothetical protein CRE_12182	−2.64	−3.10
BUX.s00460.415		−2.64	−2.46
BUX.s01513.177	Protein MLT-10	−2.64	−3.67
BUX.s00116.413	Protein MAM-3	−2.63	−2.11
BUX.s01187.48	Bovine pancreatic trypsin inhibitor domain protein	−2.63	−2.63
BUX.s00773.15		−2.63	−3.60
BUX.s00351.310	Hypothetical protein WUBG_04316	−2.62	−8.15
BUX.s00983.5	Cathepsin B-like cysteine proteinase	−2.61	−3.41
BUX.s01092.260	Hypothetical protein CAEBREN_32008	−2.61	−2.66
BUX.s01145.90		−2.61	−3.08
BUX.s00579.731	Flavin-binding monooxygenase-like family protein	−2.59	−2.73
BUX.s00294.36	Hypothetical protein WUBG_04670	−2.58	−3.43
BUX.s01147.22		−2.58	−3.50
BUX.s00713.915	Hypothetical protein CRE_18869	−2.57	−3.11
BUX.s00298.59		−2.56	−2.01
BUX.s00422.72	Protein K02E10.4, isoform a	−2.56	−2.61
BUX.s00116.766	Hypothetical protein CAEBREN_14873	−2.56	−2.10
BUX.s00351.311	Hypothetical protein LOAG_04534	−2.55	−2.20
BUX.s00540.1	Hypothetical protein CAEBREN_15368	−2.54	−2.42
BUX.s01281.22	Hypothetical protein WUBG_11029, partial	−2.54	−2.62
BUX.s01513.56	Hypothetical protein DAPPUDRAFT_223767	−2.53	−2.80
BUX.s01661.68	Hypothetical protein CRE_04031	−2.53	−2.84
BUX.s01254.375		−2.53	−2.61
BUX.s01175.26	Protein H03E18.1	−2.53	−2.22
BUX.s00725.3	Hypothetical protein CRE_04036	−2.51	−2.32
BUX.s01144.123	Protein K08B12.1	−2.51	−4.27
BUX.s00600.121	Hypothetical protein CAEBREN_16537	−2.51	−2.05
BUX.s01144.243	Protein F53B1.4	−2.51	−2.21
BUX.s01109.55	Hypothetical protein CRE_04039	−2.50	−2.07
BUX.s01513.308	Protein GRD-15	−2.50	−2.74
BUX.s00669.25	Protein DCT-5	−2.50	−2.09
BUX.s01038.225	*C. briggsae* CBR-COL-130 protein	−2.49	−2.19
BUX.s00139.93		−2.49	−2.73
BUX.c04026.1		−2.49	−3.39
BUX.s00422.270	Protein MUA-3, isoform a	−2.49	−2.52
BUX.s00351.229	Hypothetical protein CAEBREN_04806	−2.48	−3.18
BUX.s00983.2	Hypothetical protein LOAG_17799	−2.47	−2.47
BUX.s00520.28	Fatty aldehyde dehydrogenase	−2.46	−2.50
BUX.s00609.23	C. briggsae CBR-DHS-29 protein	−2.45	−2.74
BUX.s01150.18	CRE-MLT-8 protein	−2.45	−2.47
BUX.s01147.224		−2.44	−2.83
BUX.s01187.10	Protein GRD-6	−2.44	−2.52
BUX.s01656.43	Hypothetical protein CAEBREN_24487	−2.42	−2.57
BUX.s00705.56	Hypothetical protein CRE_12377	−2.42	−2.06
BUX.s00128.24	Uncharacterized protein LOC100898457	−2.42	−2.61
BUX.s01259.104		−2.41	−3.67
BUX.s01661.67	Protein NEP-17, isoform a	−2.40	−3.93
BUX.s01149.104	*C. briggsae* CBR-LET-2 protein	−2.38	−2.24
BUX.s01254.265	Hypothetical protein CRE_12586	−2.37	−3.99

Table S3. *Cont.*

Gene ID	Annotation	Change Fold in Bx_b (Fold)	Change Fold in Bx_Fungus (Fold)
BUX.s01281.86		−2.37	−2.02
BUX.s00116.457	Cytosolic glutathione S-transferase 2	−2.37	−3.35
BUX.s00351.217	Hypothetical protein LOAG_17817	−2.36	−2.79
BUX.s01281.338	Hypothetical protein LOAG_07526	−2.36	−2.01
BUX.s00422.384	Collagen alpha-3(VI) chain	−2.36	−2.48
BUX.s00422.474	Hypothetical protein LOAG_01102	−2.35	−2.38
BUX.s01144.296	Hypothetical protein CAEBREN_21159	−2.35	−2.27
BUX.s00804.3	Nuclear Hormone Receptor family member	−2.35	−2.98
BUX.s01198.107		−2.35	−3.45
BUX.s00351.421	Protein T23E7.2, isoform b	−2.35	−3.36
BUX.s01167.15		−2.34	−3.93
BUX.s01066.66		−2.34	−2.13
BUX.s01109.586	Protein COL-125	−2.33	−4.52
BUX.s00298.36	Protein W01A11.1	−2.32	−2.80
BUX.s00364.188	Collagen α-1(IV) chain	−2.32	−2.40
BUX.s00116.405	Zinc finger protein, partial	−2.32	−2.99
BUX.s00139.92		−2.30	−2.52
BUX.s00579.148	Protein COL-171	−2.30	−3.85
BUX.s01198.149	CRE-TAG-163 protein	−2.29	−2.95
BUX.s00983.4	Cathepsin B-like proteinase	−2.28	−2.34
BUX.s00773.31		−2.27	−5.16
BUX.s01268.26	Hypothetical protein CAEBREN_12945	−2.25	−3.37
BUX.s01167.13	Protein NOAH-1, isoform b	−2.25	−3.34
BUX.s01144.303	*C. briggsae* CBR-DPY-6 protein	−2.24	−5.00
BUX.s00579.210	CRE-NAS-37 protein	−2.24	−4.34
BUX.s00139.110	Protein Y53F4B.25	−2.24	−2.32
BUX.s01147.18		−2.23	−3.92
BUX.s01653.425	Hypothetical protein CBG18576	−2.23	−5.27
BUX.s00252.85	Cuticle collagen dpy-7	−2.22	−4.31
BUX.s01109.553	PAN domain-containing protein, partial	−2.21	−2.24
BUX.s01268.51		−2.20	−3.22
BUX.s00036.121		−2.19	−4.02
BUX.s01167.35	Protein C49A1.10	−2.19	−3.23
BUX.s00508.22	Hypothetical protein WUBG_08140	−2.18	−2.26
BUX.s01337.71		−2.18	−2.35
BUX.s00351.300	Hypothetical protein CRE_07231	−2.18	−2.06
BUX.s01281.370	Hypothetical protein CRE_11835	−2.18	−2.27
BUX.s01143.87	CBN-BUS-19 protein	−2.18	−2.10
BUX.s01038.251	Hypothetical protein CBG01351	−2.17	−8.06
BUX.s01513.218		−2.17	−2.49
BUX.s00579.63	Hypothetical protein CAEBREN_01577	−2.16	−2.83
BUX.c03499.1		−2.16	−2.53
BUX.s00713.55	Hypothetical protein CRE_16635	−2.16	−2.22
BUX.s01147.185	Protein T10G3.3	−2.16	−3.64
BUX.s01038.7	Hypothetical protein CRE_05305	−2.14	−2.54
BUX.s01149.42	Hypothetical protein CBG15681	−2.14	−3.00
BUX.s00351.233	DnaJ domain containing protein	−2.14	−2.26
BUX.s01660.62	Hypothetical protein WUBG_04506	−2.14	−2.58
BUX.s00466.158	Hypothetical protein Cbre_JD09.001	−2.13	−2.79
BUX.s00460.124	Protein PHG-1	−2.13	−2.12
BUX.s01259.60	Hypothetical protein CRE_28748	−2.13	−2.10
BUX.s00713.352		−2.13	−2.48
BUX.s00116.743	Hypothetical protein CBG01832	−2.13	−3.70
BUX.s00460.36	Protein COL-48	−2.13	−2.51
BUX.s01038.223	Intermediate filament protein HG-IF1	−2.12	−3.19
BUX.s00579.724	Protein BUS-17	−2.12	−3.41
BUX.s01656.64	Talin 1	−2.12	−2.10

Table S3. *Cont.*

Gene ID	Annotation	Change Fold in Bx_b (Fold)	Change Fold in Bx_Fungus (Fold)
BUX.s00116.616	Hypothetical protein CRE_10276	−2.12	−4.73
BUX.s00579.219	Hypothetical protein LOAG_05745	−2.12	−2.26
BUX.s01144.160	Nuclear Hormone Receptor family member	−2.12	−3.05
BUX.s01092.110	Hypothetical protein LOC100637862, partial	−2.12	−2.56
BUX.s00713.1153		−2.11	−3.20
BUX.s01066.173		−2.11	−3.00
BUX.s00242.1	Protein HCH-1	−2.11	−2.59
BUX.s00333.154	Hypothetical protein LOAG_12148	−2.11	−3.00
BUX.s01142.2	Collagen protein	−2.11	−3.88
BUX.s00460.53	Hypothetical protein LOAG_12928	−2.10	−4.79
BUX.s01187.44	CBN-LIPL-6 protein	−2.10	−3.15
BUX.s00351.326	Hypothetical protein LOAG_16969	−2.10	−2.51
BUX.s01109.509	Hypothetical protein CBG13113	−2.10	−2.64
BUX.s01297.1	Hypothetical protein LOAG_18663	−2.10	−2.61
BUX.s01145.15	Protein LGC-22, isoform b	−2.10	−2.34
BUX.s00116.759	*C. briggsae* CBR-PHY-2 protein	−2.10	−2.00
BUX.s01513.114	Hypothetical protein Bm1_06695	−2.09	−2.02
BUX.s01144.187	Aspartic protease 2A	−2.09	−3.84
BUX.s00579.72		−2.08	−7.59
BUX.s00713.819	2-acylglycerol O-acyltransferase 2	−2.08	−2.05
BUX.s01518.97	Protein E04D5.4, isoform a	−2.08	−3.11
BUX.s01254.23	Short-chain Dehydrogenase, partial	−2.07	−7.39
BUX.s01653.404	hypothetical protein LOAG_00505	−2.06	−2.85
BUX.s01518.84	Hypothetical protein Bm1_12890	−2.06	−2.24
BUX.s00713.927	Reductase SDR family member 1-like	−2.05	−7.17
BUX.s00116.581	Major allergen	−2.05	−5.31
BUX.s01661.59	Protein UNC-62, isoform a	−2.05	−2.25
BUX.s00579.141		−2.04	−3.83
BUX.s00578.1	Cadherin domain containing protein	−2.04	−2.15
BUX.s00813.102	Hypothetical protein CRE_00277	−2.03	−3.70
BUX.s01659.4	*C. briggsae* CBR-PCP-1.1 protein	−2.03	−2.43
BUX.c03203.1	Hypothetical protein CAEBREN_02229	−2.03	−4.29
BUX.s00128.19	Protein ASM-3, isoform a	−2.03	−2.29
BUX.s00351.416	Hypothetical protein CAEBREN_23751	−2.03	−2.33
BUX.s01143.167		−2.02	−2.93
BUX.s00333.7	Hypothetical protein CAEBREN_07108	−2.02	−2.73
BUX.s00773.30		−2.02	−3.27
BUX.s01142.36	Tetraspanin family protein	−2.01	−2.94
BUX.s00259.2	Hypothetical protein CRE_17148	−2.01	−2.51
BUX.s00460.191	Trehalose-6-phosphate hydrolase	2.00	2.28
BUX.s01147.184	Protease, serine, 22	2.02	2.05
BUX.c06670.1		2.04	2.29
BUX.s00063.1	CBN-CYP-33D1 protein	2.05	3.26
BUX.s00460.290		2.05	2.01
BUX.s01254.165		2.09	2.44
BUX.s00351.40	Thrombin inhibitor haemalin	2.11	3.29
BUX.s00302.1		2.19	2.85
BUX.s01066.133		2.22	2.22
BUX.s01089.1	CRE-STR-135 protein	2.25	3.33
BUX.s00813.54	cathepsin	2.25	2.04
BUX.s00055.336	Metallo-beta-lactamase superfamily protein	2.26	2.51
BUX.s00813.53	Cathepsin	2.30	2.20
BUX.s00647.196		2.31	2.66
BUX.s01656.34		2.39	2.25
BUX.s00036.74	Zinc metallopeptidase 2 MEP2	2.40	2.25
BUX.s01254.60	CBN-DHS-20 protein	2.46	3.85

Table S3. *Cont.*

Gene ID	Annotation	Change Fold in Bx_b (Fold)	Change Fold in Bx_Fungus (Fold)
BUX.s00116.221	CRE-TAG-89 protein	2.48	2.71
BUX.s00579.85	Venom allergen-like protein VAP1	2.49	2.73
BUX.s00647.87		2.54	3.89
BUX.s01143.152	Histone-lysine N-methyltransferase SETMAR	2.59	2.97
BUX.s00333.185	CRE-SRH-276 protein	2.65	2.76
BUX.s01661.72	Protein NEP-17, isoform a	2.77	2.47
BUX.s00713.60	PHD finger-like domain protein 5A	2.93	3.33
BUX.s01144.145	Protein SRH-264	3.00	3.36
BUX.s00713.1015		3.00	4.00
BUX.s00813.52	Cathepsin	3.12	2.42
BUX.s01145.123		3.21	2.63
BUX.s00364.261	Hemoglobinase-type cysteine proteinase	3.25	3.65
BUX.s01337.81		3.38	2.54
BUX.s00647.111	Glutathione *S*-transferase S1	3.39	2.49
BUX.s00813.51	Cathepsin	3.62	2.24
BUX.s00647.197		3.71	4.60
BUX.s01044.1		5.50	6.50
BUX.s00713.1054		6.00	5.50

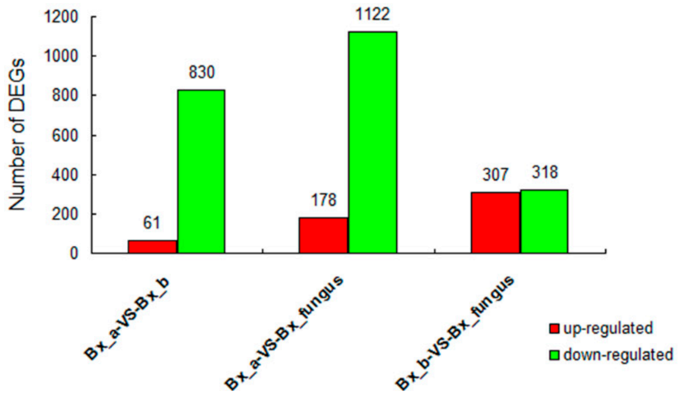

Figure S1. Genes most differentially expressed in the three different treated samples of *B. xylophilus*. Bx_a: aseptic *B. xylophilus*; Bx_b: *B. xylophilus* treated with *S. maltophilia* NSPmBx03; Bx_fungus: fungus *B. xylophilus*.
